# Supplementary material for: The evolutionary plasticity of chromosome metabolism allows adaptation to constitutive DNA replication stress
Source: eLife. 2020 Feb 11;9:e51963. doi: 10.7554/eLife.51963 (PMC7069727; doi:10.7554/eLife.51963)
Supplement: Supplementary file 6. [file elife-51963-supp6.docx]

**Tables represented in figures**

**Figure 1-Figure supplement 2**

| clone | gene | nucleotide change | AA and regulatory  change | segregation |
| --- | --- | --- | --- | --- |
| EVO1-7 | *IXR1* | 1393 A→C | T465P | 97% |
|  | *RAD9* | 3286 G→A | G1096E | 74% |
|  | *TIR1* | 426-464del | 139-188del | 91% |
| EVO2-10 | *PSF3* | 134 G→A | S45N | 88% |
|  | *NVJ2* | -559 G→C | promoter | 84% |
| EVO3-12 | *SIR4* | 1877 C→A | S626* | 97% |
|  | *IXR1* | 922 C→T | Q308* | 94% |
|  | *MMS1* | 2170 G→T | A724S | 88% |
|  | *DPB11* | 1804 +A | P601* | 76% |
|  | *RPS28B* | 42 -G | terminator | 90% |
| EVO4-2 | *IXR1* | 79 C→T | Q27* | 80% |
|  | *SIR4* | 3140 C→T | S1047F | 95% |
|  | *RAD9* | 2628 +A | N876K | 81% |
|  | *RPS28B* | 42 -G | terminator | 76% |
|  | *CDD1* | 68 +TTTT | terminator | 73% |
| EVO5-11 | *SLD5* | 388 G→A | E130K | 83% |
|  | *CTH1* | 4 A→G | M2V | 71% |
|  | *GIR2* | -197 T→C | promoter | 71% |
| EVO6-1 | *IXR1* | 1263 C→G | Y421* | 94% |
|  | *SIR3* | 32 G→A | W11* | 79% |
|  | *SMC2* | 940 C→A | R164S | 71% |
|  | *UTR2* | -524 C→T | promoter | 80% |
| EVO7-7 | *PSF3* | 53 G→T | C18F | 93% |
|  | *CTR9* | 976 T→A | L326I | 84% |
|  | *DSF2* | 772 +A | T258L | 84% |
| EVO8-9 | *PSF1* | 599 T→A | I200N | 82% |

**Figure 2B**

| Gene | Population hits | % Mutant  reads | Role |
| --- | --- | --- | --- |
| *RAD9* | 4 | 63.80% | Mediator |
| *MEC1* | 1 | 18% | Sensor kinase |
| *TEL1* | 1 | 64% | Sensor kinase |
| *LCD1* | 1 | 62.50% | Mec1 binding partner |
| *DPB11* | 1 | 97% | Sensor |

**Figure 2-Figure supplement 1A**

| Gene | Unique  hits | Nucleotide  change | AA  change | Type | Note |
| --- | --- | --- | --- | --- | --- |
| *RAD9* | 5 | 2628 +A | frameshift | indel | K883* |
| *RAD9* | 1 | 3017 T→G | L1006W | substitution |  |
| *RAD9* | 1 | 1904 +A | frameshift | indel | D638* |
| *RAD9* | 1 | 3287 G→A | G1096E | substitution |  |
| *RAD9* | 1 | 3835 G→A | E1278K | substitution |  |
| *MEC1* | 1 | 3917 C→T | A1306V | substitution |  |
| *TEL1* | 1 | 2282 C→A | T2028K | substitution | kinase domain |
| *LCD1* | 1 | 536 G→A | R179H | substitution |  |
| *DPB11* | 1 | 1804 +A | frameshift | indel | S608* |

**Figure 3B**

| Gene | Populations  hits | % Mutant  reads | Role |
| --- | --- | --- | --- |
| *RAD61* | 1 | 82% | Cohesin dynamics |
| *CHL1* | 1 | <10% | Non essential cohesion factor |
| *PDS5* | 1 | <10% | Cohesin dynamics |
| *SMC2* | 1 | <10% | Condensin complex |
| *CSM3* | 1 | <10% | Non essential cohesion factor |
| *TOF1* | 1 | <10% | Non essential cohesion factor |

**Figure 3-Figure supplement 2A**

| Gene | Unique  hits | Nucleotide  change | AA  change | Type | Note |
| --- | --- | --- | --- | --- | --- |
| *RAD61* | 1 | 2628 T→A | Promoter | substitution |  |
| *CHL1* | 1 | 2050 G→A | D684N | substitution | helicase domain |
| *PDS5* | 1 | 204 A→C | K68N | substitution |  |
| *SMC2* | 1 | 940 C→A | R314S | substitution |  |
| *TOF1* | 1 | 1244 C→A | P415Q | substitution |  |
| *CSM3* | 1 | 370 G→C | V124L | substitution |  |

**Figure 4A**

| Gene | Population hits | % mutant  reads | Role |
| --- | --- | --- | --- |
| *IXR1* | 6 | 98% | dNTPs TF |
| *PSF3* | 2 | 100% | GINS |
| *TOP1* | 2 | 27% | Topoisomerase |
| *DPB2* | 2 | 56% | DNA polymerase |
| *PSF1* | 1 | 82% | GINS |
| *DPB11* | 1 | 97% | Replication initiation |
| *SLD5* | 1 | 10% | GINS |
| *CHL1* | 1 | <10% | Okazaki fragment processing |
| *RFC1* | 1 | <10% | Polymerase clamp loader |
| *TOF1* | 1 | <10% | Fork protection complex |
| *CSM3* | 1 | <10% | Fork protection complex |

**Figure 4-Figure supplement 1A**

| Gene | Unique hits | Nucleotide  change | AA  change | Type | Note |
| --- | --- | --- | --- | --- | --- |
| *IXR1* | 1 | 1393 A→C | T465P | substitution | HMG domain |
| *IXR1* | 1 | 922 C→T | Q307* | substitution |  |
| *IXR1* | 1 | 79 C→T | Q27* | substitution |  |
| *IXR1* | 1 | 1263 C→G | Y421* | substitution |  |
| *IXR1* | 1 | 1075 -C | Q359K | indel | R363* |
| *IXR1* | 1 | 994 C→T | Q332* | substitution |  |
| *PSF3* | 1 | 569 G→A | W190* | substitution |  |
| *PSF3* | 1 | 53 G→T | C18F | substitution |  |
| *TOP1* | 1 | -243 G→A | promoter | substitution |  |
| *TOP1* | 1 | 1257 A→T | L419F | substitution | catalytic core |
| *DPB2* | 1 | 64 T→C | Y22H | substitution |  |
| *DPB2* | 1 | 1064 C→T | T355I | substitution |  |
| *PSF1* | 1 | 599 T→A | I200N | substitution |  |
| *DPB11* | 1 | 1804 +A | P602* | substitution |  |
| *SLD5* | 1 | 388 G→A | E130K | substitution |  |
| *CHL1* | 1 | 2050 G→A | D684N | substitution | helicase domain |
| *RFC1* | 1 | -156 +A | promoter | indel |  |
| *TOF1* | 1 | 1244 C→A | P415Q | substitution |  |
| *CSM3* | 1 | 370 G→C | V124L | substitution |  |
